# Describing the Reproductive Microbiome of *Tritrichomonas foetus* Chronically Infected Bulls and Diagnostic Collection Device Performance

**DOI:** 10.3390/ani14182689

**Published:** 2024-09-16

**Authors:** SaraBeth Boggan, Babafela Awosile, Jennifer Koziol

**Affiliations:** School of Veterinary Medicine, Texas Tech University, 7671 Evans Drive, Amarillo, TX 79106, USA; sboggan@ttu.edu (S.B.); babafela.awosile@ttu.edu (B.A.)

**Keywords:** *Tritrichomonas foetus*, bovine *tritrichomonas*, microbiome, penile microbiome, preputial microbiome, seminal microbiome, cattle reproductive health, bacterial diversity, protozoan parasite, sampling methods

## Abstract

**Simple Summary:**

This study investigates the microbiome of bulls infected with *Tritrichomonas foetus* (*T. foetus*), a protozoan causing bovine tritrichomoniasis. It aims to describe the preputial, penile, and seminal microbiomes of chronically infected bulls and evaluate different sampling methods. Eleven bulls were sampled using dacron swabs, pizzle sticks, double-guarded swabs, and semen collection. The microbiomes were dominated by Fusobacteria, Firmicutes, Bacteroidota, Actinobacteria, and Campylobacterota, with semen samples showing the most microbial diversity. Despite differences in operational taxonomic units (OTUs) between methods, there was no significant difference in microbial richness and abundance. The study highlights that *T. foetus* infections may lead to shifts in the reproductive microbiome, indicating a potential dysbiosis compared to healthy bulls. These findings provide a basis for future research on *T. foetus* impacts on bovine reproductive health and possible treatment strategies.

**Abstract:**

*Tritrichomonas foetus (T. foetus)*, the causative agent of bovine trichomoniasis, is an obligate protozoan parasite of the bovine reproductive tract and can be found on the penis, prepuce, and distal urethra of the bull and from the cranial vagina to the oviduct in the infected cow. To date, the microbiome of bulls infected with *T. foetus* has not been described. The objectives of this study were to (1) describe the preputial and penile microbiome of bulls chronically infected by *T. foetus,* (2) describe the seminal microbiome of *T. foetus*-infected bulls, and (3) evaluate different collection devices that could be used for sampling. Eleven bulls naturally infected with *T. foetus* were utilized for the collection of samples. Samples were obtained during the process of a routine breeding soundness exam utilizing either a dacron swab, pizzle stick, double-guarded swab, or semen collection. The preputial and seminal microbiome of *T. foetus*-infected bulls was dominated by bacterial members of the phyla *Fusobacteriota, Firmicutes*, *Bacteroidota, Actinobacteria,* and *Campylobacterota.* Semen collection yielded the most microbial diversity; however, there was no significant difference between the four methods (*p* ≥ 0.05). This study characterizes both the preputial and seminal microbial communities of bulls chronically infected by *T. foetus.*

## 1. Introduction

*Tritrichomonas foetus*, the causative agent of bovine trichomoniasis, is an obligate protozoan parasite of the bovine reproductive tract and is one of two true venereal diseases in cattle in that the disease is only spread via coitus and only affects the reproductive tract of infected animals. *T. foetus* can be found on the penis, prepuce, and distal urethra of the bull and from the level of the cranial vagina to the oviduct in the infected cow. Bulls experience no clinical signs associated with infection and are considered chronically infected as they cannot mount an immune response sufficient to clear the infection [1,2,3]. To date, the microbiome of bulls infected with *T. foetus* has not been described. This work will aid in future exploration of therapies that can improve or eliminate disease risk in cattle.

Humans can be infected with *Trichomonas vaginalis,* a related organism to *T. foetus* that is the most common non-viral sexually transmitted infection (STI). Due to the frequent nature of this disease in humans, it has been extensively studied. Researchers have recently begun investigating the effect of the microbiome on disease status, and they have characterized the microbiome of *T. vaginalis* infection. This knowledge has been used to help researchers understand the risk of sexually transmitted infection (STI) contraction and development of the carrier status [4,5]. Understanding shifts in the human microbiome has shown to be critical in the exploration of vaccines and antiprotozoal therapies, aiding in the stabilization of the reproductive microbiome in the hope of decreasing the risk of contracting STIs [6].

As of 2022, the United States cattle industry accounts for approximately 17% of the USD 462 billion agriculture economy, with an estimated 91.9 million head of cattle in the country [7]. Given the size of this industry, cattle producers work to ensure safe and efficient production. One aspect of this efficient production is the reproductive health and success of cattle herds. When not carefully managed, there are many diseases that can have devastating impacts on the reproductive capabilities of a cattle herd with long-lasting economic impacts on the producers; diseases such as brucellosis, leptospirosis, infectious bovine rhinotracheitis, bovine viral diarrhea, and *T. foetus* are a few [8].

Cows become infected with *T. foetus* following coitus with an infected bull, where mechanical transmission occurs from the penis and prepuce to the vaginal vault. This infection results in endometritis, cervicitis, and vaginitis. This infection frequently leads to embryonic death, abortion, pyometra, and fetal maceration. These are conditions that contribute to the major economic losses for cattle producers, including calf crop loss secondary to embryonic loss or abortion, reduced weaning weights due to delayed conception, and culling and replacement of infected cattle due to the lack of legal treatment. Infected bulls are asymptomatic carriers of *T. foetus* and transmit the organism to cows during coitus [9]. While no negative health-related side effects are documented from the infection in bulls, little is known about the impact(s) on the presence of *T. foetus* on the bovine penile and preputial microbiomes or the seminal microbiome.

Microbiomes provide a wide range of information, ranging from assessing human and animal health, evaluating environmental changes, to providing insights on improving agriculture practices [10]. In previous years, the vaginal and uterine microbiomes of cows have been described [11,12,13,14,15], and in a 2020 study, the preputial microbial composition of healthy bulls was described [16].

Understanding the impacts of *T. foetus* on the reproductive microbiome of bulls could lead to greater understandings of potential measures for *T. foetus* management. The objectives of this study were to (1) describe the preputial/penile microbiome of bulls chronically infected by *Tritrichomonas foetus*, (2) describe the seminal microbiome of bulls chronically infected by *Tritrichomonas foetus,* and (3) evaluate different collection devices that could be used for sampling.

## 2. Materials and Methods

### 2.1. Animals and Sample Collection

Eleven bulls naturally infected with *T. foetus* were included in this study in a descriptive epidemiological study of the preputial and semen microbiome. The bulls included in this study were between 2 and 6 years of age and consisted of Hereford, Angus, and Charolais breeding. All bulls were housed in the same dirt paddock and fed a total mixed ration in Bushland, Texas. The Texas Tech University animal care and use program is accredited by AAALAC International, and the Texas Tech Institutional Animal Care and Use Committee (IACUC) reviewed and approved this protocol (2022-1177). This study is compliant with current ARRIVE guidelines.

Bulls were restrained in a livestock squeeze chute per routine standards for performing routine breeding soundness examinations. Four different sample collection methods (dacron swab, pizzle stick, guarded swab, and semen) were used in the collection of preputial and semen samples from the 11 bulls for the microbiome study. Swab samples were obtained during the process of a routine breeding soundness exam as previously described in Wickware et al. utilizing a dacron swab and will be referred to as dacron swabs from here on out [16]. Only 8 bulls were swabbed with dacron swabs due to bull availability on that day. Additionally, preputial samples were obtained utilizing the Pizzle Stick Trich testing device (pizzle stick), a specially designed *T. foetus* testing apparatus (Lane Manufacturing, Denver, CO, USA), and a double-guarded 33-inch culture swab (guarded swab) (Jorgenson Laboratories, Loveland, CO, USA) with a minimum of 7 days between testing. All samples were taken from bulls in the same order and without randomization. Lastly, semen was also collected from these bulls in the same manner as Koziol et al. [17]. For all sample collection procedures, proper hygiene, aseptic handling, and personal protective equipment were utilized.

Dacron swabs, pizzle stick samples, and guarded swabs were immediately placed in 3 mL sterile PBS and placed on ice. Semen samples were collected into cryovials, which were also immediately placed on ice following collection. Samples were then transported to the laboratory and stored at −80 °C until processed for DNA extraction.

### 2.2. DNA Extraction and 16S Sequencing

DNA was extracted from samples with the MagMax DNA Multi-Sample Ultra 2.0 Kit (Thermo Fisher Scientific Inc., Waltham, MA, USA) using the automated KingFisher Apex (Thermo Fisher Scientific) instrument, following the manufacturer’s protocol. Aliquots of each sample were taken and placed into clean 96-well plates by adding lysis buffer to each sample and vortexing the plate for three minutes. Then isopropanol was added and shook for an additional three minutes. At this point, DNA-binding beads were added; the samples were shaken again and placed on a magnetic stand to pellet the beads. Then the samples were washed with two wash solutions. The DNA was then eluted by adding elution buffer and vortexing, then transferring the purified DNA into clean wells. A detailed protocol can be found described as previously described by McLaughlin, 2021 [18]. DNA concentration and purity were monitored on 1% agarose gels. According to the concentration, DNA was diluted to 1 ng/μL using sterile water. Briefly, Illumina-indexed reads were created using PCR amplification of the V3–V4 region of bacterial 16S rRNA gene using the 341F (CCTAYGGGRBGCASCAG)/806R (GGACTACNNGGGTATCTAAT) [19]. Thermal cycling consisted of initial denaturation at 98 °C for 1 min, followed by 30 cycles of denaturation at 98 °C for 10 s, annealing at 50 °C for 30 s, and elongation at 72 °C for 30 s. Finally, 72 °C for 5 min. PCR-grade water was used as an amplification negative control and a mock community (20 Strain Even Mix 138 Genomic Material; ATCC^®^ MSA-1002TM) as positive control. Amplification success was determined using 2% gel electrophoresis as a quality check, and no bands were observed in the negative control. Amplified PCR products were mixed in equidensity ratios and then purified with the Universal DNA Purification Kit (TianGen, Beijing, China, Cat# DP214). The pooled amplification products from preputial samples, mock community, and water were sequenced (Illumina NovaSeq 6000, paired end 250 bp, 100 kb raw reads) by Novogene (Cambridge, UK). Extracted and quantified DNA was used for the construction of a 16S rRNA gene library following a standardized protocol [19]. Raw reads (5,850,481 read pairs from 30 samples) were analyzed, and sequencing libraries were generated using the NEB Next^®^ Ultra™ II FS DNA PCR-free Library Prep Kit (New England Biolabs, Ipswich, MA, USA, Catalog #: E7430L) following manufacturers recommendations.

The library quality was assessed with a Qubit @ 2.0 fluorometer (Thermo Scientific) and real-time PCR for quantification and an Agilent Bioanalyzer 2100 system for size distribution detection. Quantified libraries were pooled and sequenced on the Illumina NovaSeq platform, and 250 bp paired-end reads were generated.

### 2.3. Statistical Analysis

For microbiome data, relative abundances for the total reads for the identified operational taxonomic units (OTUs), alpha, and beta diversity were calculated using QIIME version 1.9.1 [20] and analyzed with R statistical software (R Core Team, 2020; Vienna, Austria, v 4.0.3). Relative abundance of microbiome data was presented using bar plots. The alpha diversity index was calculated using the alpha_diversity.py script of the QIIME software. The default parameters of QIIME software (Version 1.9.1) were used to set the minimum number of extracted sequences as 10, the maximum number of extracted sequences as the minimum number of corresponding sequences among all samples, and the step size as (maximum number of extracted—minimum number of extracted)/10, with 10 repetitions of sampling in each step. The number of reads chosen for normalization cutoff was 92,244. The cutoff was used to rarefy the microbiome data. We assessed alpha diversity using Shannon’s diversity index as a measure of microbial species richness and evenness and Chao’s richness estimate as a measure of uniqueness of OTU. We compared the relative abundance, and alpha diversity indices between the sample groups using the Kruskal–Wallis test. Beta diversity of the microbiome data between the sample groups was evaluated using weighted and unweighted Unifrac distance as calculated by QIIME software [21,22]. The beta diversity between the sample groups was visualized using the nonmetric multi-dimensional scaling (NMDS) ordination plot. We analyzed the variation and differences in distance of beta diversity between the sample groups using the analysis of similarity (ANOSIM). We used similarity percentage (SIMPER) analysis to explore the specific OTU driving differential abundance of the microbial community between the sample groups.

## 3. Results

**Figure 1:**
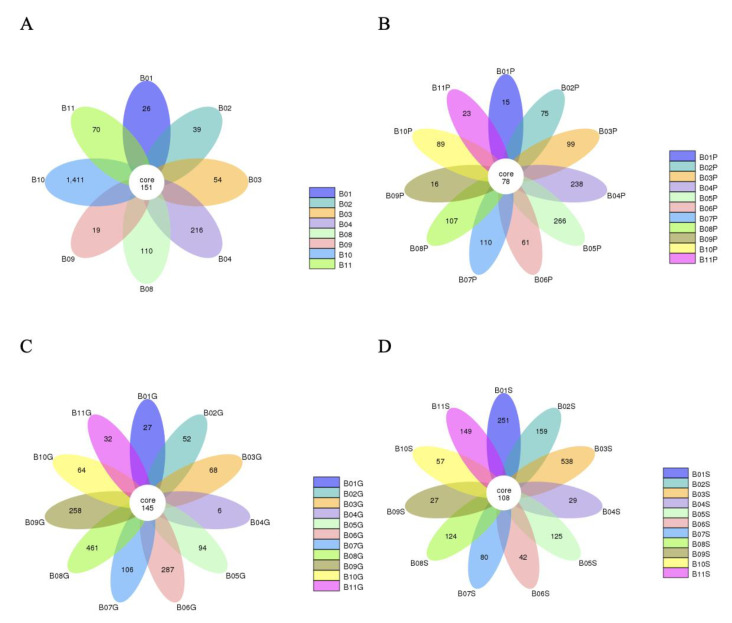
Flower diagrams showing the number of OTUs unique per bull as well as the OTUs shared by all bulls per sampling method. (**A**) OTUs obtained via dacron swabs, (**B**) pizzle sticks, (**C**) guarded swabs, (**D**) semen samples.

A total of 92,244 read sequences were generated following next-generation sequencing for the expression of bacterial 16S rRNA genes. Using dacron swabs, 2096 operational taxonomic units (OTUs) were recovered from 8 bulls, 1600 OTUs were obtained from 11 bulls via guarded swabs, 1177 OTUs were obtained from 11 bulls via pizzle sticks, and 1689 OTUs were obtained from 11 bulls via semen samples (Figure 1). Of the 482 core OTUs obtained across the various sampling methods, 79 were only obtained from their respective sampling method and not repeated via another sampling method. When the duplicate OTUs were removed across the different sampling methods, a total of 4342 unique OTUs were identified in this study.

**Figure 2:**
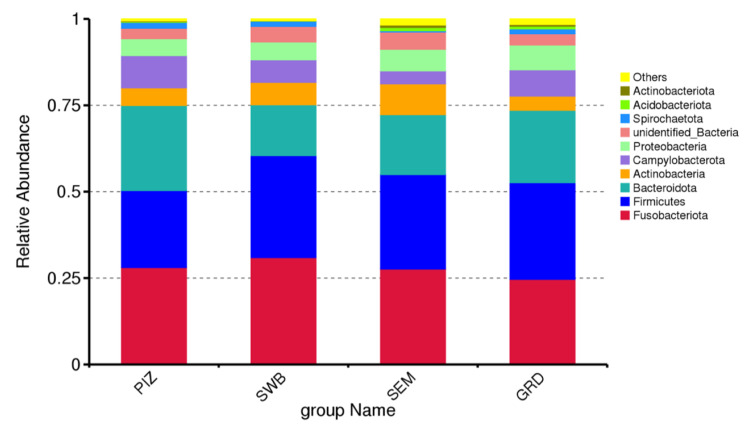
Relative abundance of top 10 bacteria phyla across all study bulls from all sampling methods. “PIZ” = pizzle sticks, “SWB” = dacron swabs, “SEM” = semen samples, and “GRD” = guarded swabs. Fusobacteriota (mean% ± SD: 27.82 ± 0.02), Firmicutes (mean ± SD: 26.78 ± 0.03), Bacteroidota (mean% ± SD: 19.41 ± 0.04), Actinobacteria (mean% ± SD: 6.15 ± 0.02), and Campylobacterota (mean% ± SD: 6.80 ± 0.02).

The preputial and seminal microbiome of *T. foetus*-infected bulls was dominated by bacterial members of the phyla *Fusobacteriota* (mean% ± SD: 27.82 ± 0.02), *Firmicutes* (mean ± SD: 26.78 ± 0.03), *Bacteroidota* (mean% ± SD: 19.41 ± 0.04), *Actinobacteria* (mean% ± SD: 6.15 ± 0.02), and *Campylobacterota* (mean% ± SD: 6.80 ± 0.02) (Figure 2). The most common genera among the infected bull samples were *Fusobacterium* (mean% ± SD: 16.86 ± 0.01), *Porphyromonas* (mean% ± SD: 12.74 ± 0.02), *Corynebacterium* (mean% ± SD: 4.46 ± 0.06), *Campylobacter* (mean% ± SD: 6.79 ± 0.02)*,* and *Streptobacillus* (mean% ± SD: 1.63 ± 0.01). The most common species among the infected bull samples were *Campylobacter ureolyticus* (mean% ± SD: 5.68 ± 0.02), *Corynebacterium pilosum* (mean% ± SD: 2.35 ± 0.01), *Bacteroidales bacterium KA00251* (mean% ± SD: 1.59 ± 0.003), *Mycoplasma feliminutum* (mean% ± SD: 1.28 ± 0.004), and *Clostridiales bacterium feline oral taxon 339* (mean% ± SD: 1.24 ± 0.005). Then, using a *T*-test, we were able to determine species that had significant variation between the groups. Testing between the guarded swab and the dacron swab, there were 10 significantly variant species, between the pizzle stick and the guarded swab, 16 species, and between the dacron swab and the pizzle stick, 14 species. The results are shown in Table 1, Table 2 and Table 3.

**Figure 3:**
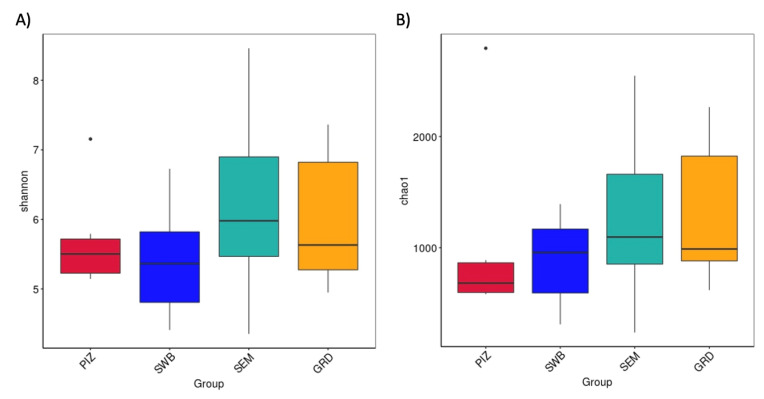
(**A**) Shannon index showing microbial species diversity per sampling method, accounting for both abundance and evenness. (**B**) Chao1 estimated species richness across all sampling methods. No significant difference shown in diversity or richness across the methods. “PIZ” = pizzle stick, “SWB” = dacron swab, “SEM” = semen, and “GRD” = guarded swab.

**Figure 4:**
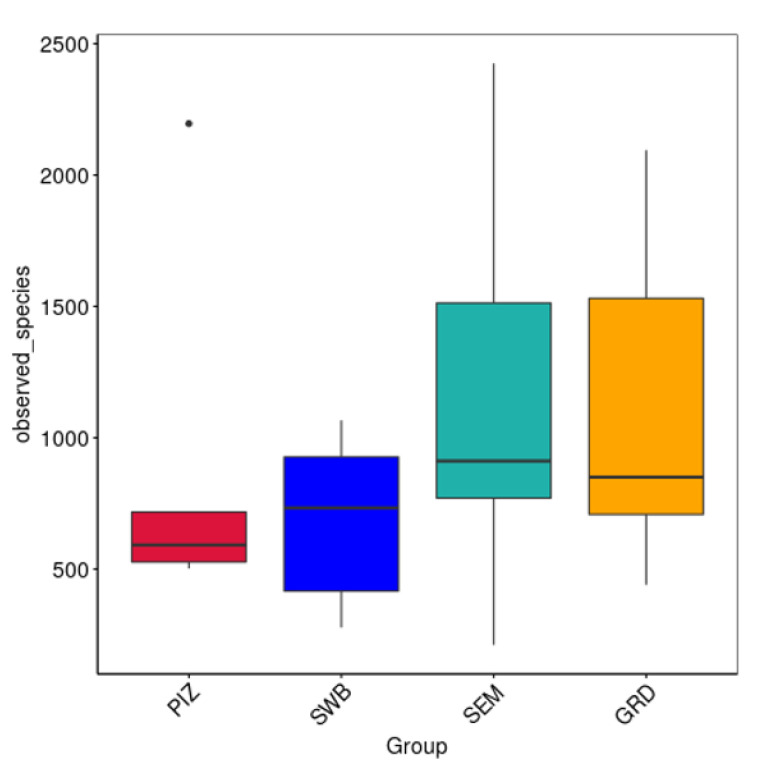
Number of species observed per sample. Represents the discovered microbial community within each sample. No significant difference between methods. “PIZ” = pizzle stick, “SWB” = dacron swab, “SEM” = semen, and “GRD” = guarded swab. Average bacterial species observed per group: PIZ, 680 ± 286.08; SWB, 796 ± 571.88; SEM, 1124 ± 701.79; GRD, 1079 ± 587.85.

The average Shannon diversity index among the bulls is 5.789 ± 0.382. The Shannon diversity index was higher in semen collection compared to other methods; however, there was no statistically significant difference (*p* > 0.05) in the measure of microbial richness and abundance between the four methods (Figure 3A). The average Chao1 estimated species richness among the bulls is 1094.09 ± 169.83 OTUs; there was no statistically significant difference between the four methods (*p* ≥ 0.05) in term of the measure of microbial richness (Figure 3B). The average number of bacterial species among the bulls is 3122.5 ± 1714.5. The average number observed per group were as follows: pizzle stick, 680 ± 286.08; dacron swab, 680 ± 571.88; semen, 1124 ± 701.79; and guarded swab, 1079 ± 587.85 (Figure 4).

**Figure 5:**
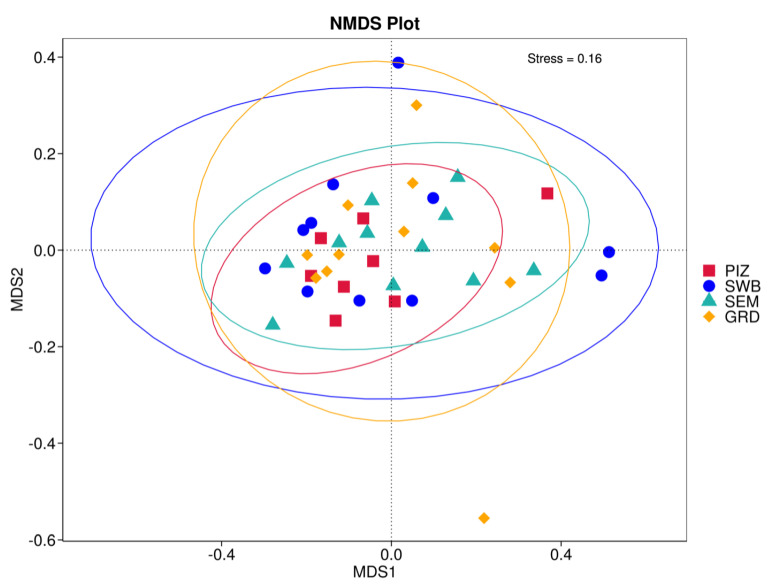
Bray–Curtis non-metric multi-dimensional scaling (NMDS) plot showing the microbial variation between samples. “PIZ” = pizzle stick, “SWB” = dacron swab, “SEM” = semen, and “GRD” = guarded swab. The samples of each group are relatively clustered, meaning there is low bacterial compositional dissimilarity.

**Figure 6:**
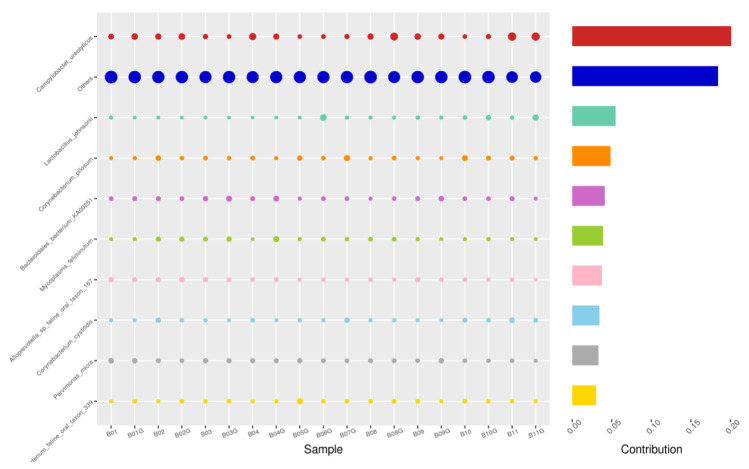
SWB-GRD, Top 10 species in terms of contribution. Vertical axis the individual bacterial species. Horizontal axis is sample, samples ending in “G” = guarded swab, all other samples were collected with the dacron swab. Contribution is measured in %. The bubble size represents the relative abundance of the species, and the contribution is the contribution of the species in the two groups of variability.

**Figure 7:**
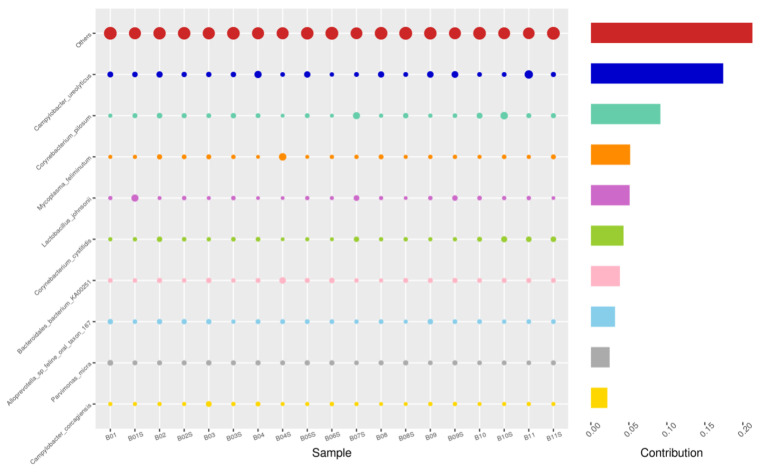
SWB-SEM, Top 10 species in terms of contribution. Vertical axis the individual bacterial species. Horizontal axis is sample, samples ending in “S” = semen, all other samples were collected with the dacron swab. Contribution is measured in %. The bubble size represents the relative abundance of the species, and the contribution is the contribution of the species in the two groups of variability.

**Figure 8:**
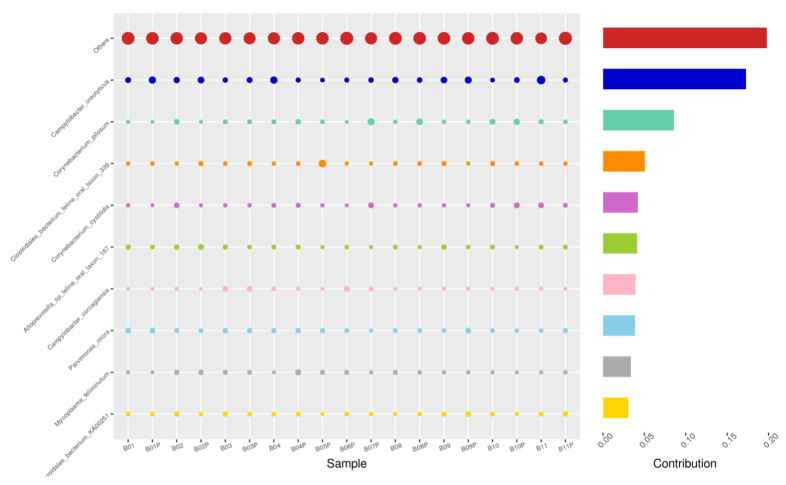
PIZ-SWB, Top 10 species in terms of contribution. Vertical axis the individual bacterial species. Horizontal axis is sample, samples ending in “P” = pizzle stick, all other samples were collected with the dacron swab. Contribution is measured in %. The bubble size represents the relative abundance of the species, and the contribution is the contribution of the species in the two groups of variability.

**Figure 9:**
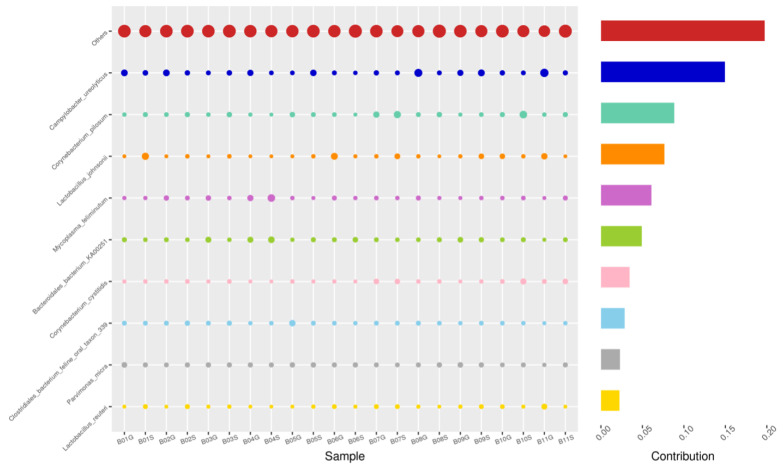
SEM-GRD, Top 10 species in terms of contribution. Vertical axis the individual bacterial species. Horizontal axis is sample, samples ending in “G” = guarded swab, “S” = semen. Contribution is measured in %. The bubble size represents the relative abundance of the species, and the contribution is the contribution of the species in the two groups of variability.

**Figure 10:**
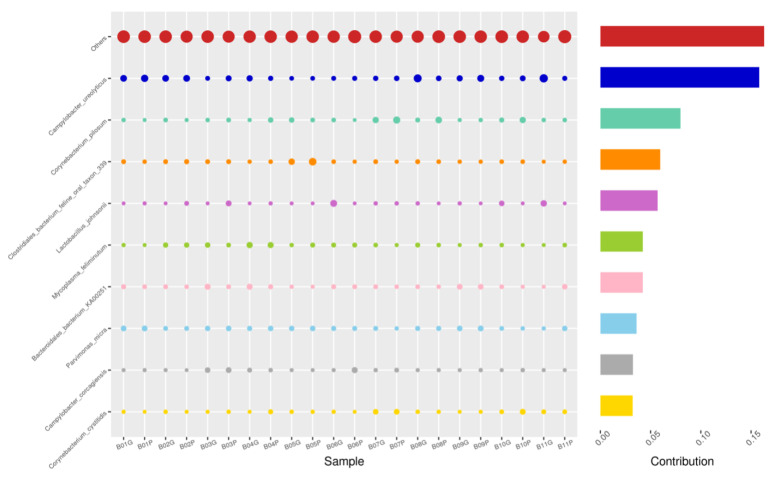
PIZ-GRD, Top 10 species in terms of contribution. Vertical axis the individual bacterial species. Horizontal axis is sample, samples ending in “G” = guarded swab, “P” = pizzle stick. Contribution is measured in %. The bubble size represents the relative abundance of the species, and the contribution is the contribution of the species in the two groups of variability.

**Figure 11:**
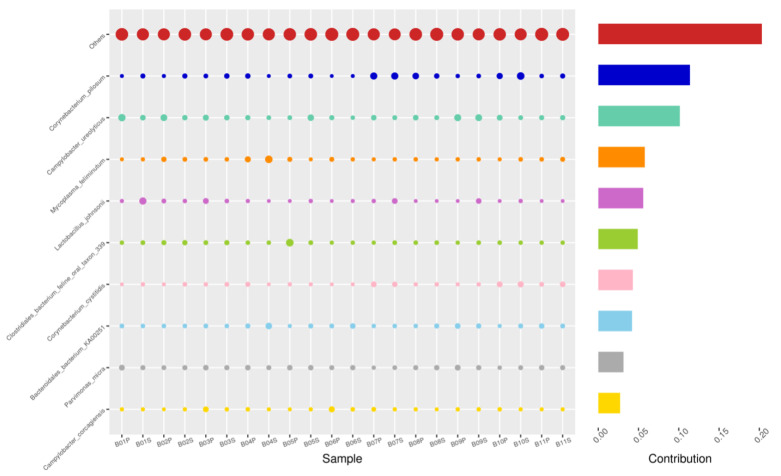
PIZ-SEM, Top 10 species in terms of contribution. Vertical axis the individual bacterial species. Horizontal axis is sample, samples ending in “P” = pizzle stick, “S” = semen. Contribution is measured in %. The bubble size represents the relative abundance of the species, and the contribution is the contribution of the species in the two groups of variability.

Using the Bray–Curtis NMDS plot for beta diversity, the groups are relatively clustered, showing. relatively low bacterial compositional dissimilarity (Figure 5). From the ANOSIM, there was no statistically significant variation in microbial beta diversity between the groups; therefore, the microbial beta diversity between the sample groups is similar. Using similarity percentage analysis (SIMPER), with which we evaluated the relative abundance of the top 10 species and the contribution of the species to the differential abundance in microbiome between testing groups. When comparing the groups against each other, *Camplobacter ureolyticus* was differentially more abundant (11.4–20.1%) than other microbiota species in all comparison groups, except in the pizzle stick verses guarded swab group, where *Corynebacterium pilosum* was differentially more abundant by 11.4% contribution, followed by *Camplobacter ureolyticus* at 10.1% (Figure 6, Figure 7, Figure 8, Figure 9, Figure 10 and Figure 11).

## 4. Discussion

The next-generation sequencing (NGS) data generated in this study provided a robust overview of the microbial diversity present in the preputial and seminal samples of *T. foetus*-infected bulls. With a total of 92,244 read sequences obtained, the study identified 4342 unique operational taxonomic units (OTUs) across all sampling methods, underscoring the richness and complexity of the microbial communities.

The dominance of certain bacterial phyla, such as *Fusobacteriota, Firmicutes*, and *Bacteroido*ta, along with the presence of key genera like *Fusobacterium*, *Porphyromonas*, and *Corynebacterium*, reflects a consistent microbial signature in *T. foetus*-infected bulls. However, the variations in species abundance between different sampling methods, as revealed by *T*-tests, suggest that while the overall microbial community structure remains relatively stable, the fine-scale composition may be influenced by the choice of sampling technique. This finding is further supported by the Shannon diversity index, which, although slightly higher in semen samples, did not show significant differences between methods, indicating that all methods are effective in capturing a broad spectrum of microbial diversity. The lack of significant differences in beta diversity, as shown by the Bray–Curtis NMDS plot and ANOSIM results, further reinforces the notion that the microbial communities are relatively similar across sampling methods. However, the differential abundance of species such as *Campylobacter ureolyticus* and *Corynebacterium pilosum* in certain comparisons highlights the importance of considering specific bacterial taxa when interpreting the results of microbiome studies in *T. foetus*-infected bulls.

Our study underscores the complexity and variability of microbial communities associated with bulls infected with *T. foetus.* The use of different sampling techniques, such as the pizzle stick, dacron swab, semen collection, and guarded swab, allowed for a comprehensive analysis of these communities within the infected bulls. Despite the variety of methods, the results indicated that the choice of sampling technique did not significantly affect the overall microbial diversity detected. We detected unique OTUs using all four sampling methods: the pizzle stick, dacron swab, semen, and guarded swab. From the results seen in our study, there was no statistically significant difference between the sampling method chosen to obtain the microbiome sample (Figure 3A,B and Figure 4). As our sample size is small, we cannot suggest that there is any benefit of one collection device over the other. However, these findings suggest that the microbial community structure remains consistent across different sampling methods, potentially providing flexibility in field studies where certain sampling tools might be more accessible or practical. Additionally, it should be noted that while a significant number of OTUs were shared across the different sampling methods, 79 OTUs were uniquely associated with their respective sampling method, highlighting the potential influence of the sampling technique on microbial detection, yet there was no statistical significance. These unique OTUs could represent niche-specific bacteria that are differentially accessed by each sampling method, suggesting that a comprehensive analysis of the bull microbiome may require a multi-method approach to capture the full diversity.

Interestingly, the dacron swab, a method previously employed in studies of healthy bulls, revealed a significantly higher number of operational taxonomic units (OTUs) in the *T. foetus*-positive bulls in our study compared to those reported in earlier studies on healthy bulls. This increase in OTUs suggests a more complex microbial environment in infected bulls, possibly due to the pathogen’s influence on the microbial landscape. The absence of documented microbiome studies utilizing the pizzle stick or guarded swab highlights a gap in the literature that our study begins to fill, providing new insights into the microbial diversity of the bull reproductive tract under different health conditions.

Moreover, the comparison between our findings and those of previous studies on healthy bulls reveals both shared and distinct microbial taxa, emphasizing the potential impact of *T. foetus* infection on the microbial composition. In a 2022 study, Koziol et al. evaluated the seminal microbiota of 45 healthy beef bulls [17]. In the Koziol et al. study, healthy bulls that were declared unsatisfactory as potential breeders shared microbiome similarities compared to *T. foetus*-positive bulls in our current study. The presence of common phyla such as *Firmicutes*, *Bacteroidota*, and *Actinobacteria* across different studies suggests a core microbiome, while the differences in genera point to microbial shifts that may be associated with infection. However, these same phyla were also detected and shared as the most common phyla in Wickware et al.’s study with the healthy bulls’ preputial microbiome [16]. Similar phyla were also noted by Cojkic et al. [23]. These shifts, potentially influenced by geographical factors, highlight the complexity of host–microbe interactions and the need for further research to elucidate the specific mechanisms driving these changes.

When compared to the Wickware et al. study of the healthy bull prepuce microbiome, our study shared some taxonomic similarities, but there were also distinct differences. When comparing the top 30 genera of the non-infected bull microbiomes and the infected bull microbiomes from our study, *Porphyromonas*, *Streptobacillus*, *Corynebacterium*, *Campylobacter*, *Mycoplasma*, *Prevotella*, *Bacillus*, *Parvimonas*, *Histophilus*, *Gemella*, and *Fusobacterium* are the only shared genera. These findings of dissimilarities between the microbiome communities suggest that bulls infected with *T. foetus* may experience a microbial shift; however, direct comparison groups would have to be made to confirm that hypothesis. The authors cannot rule out differences due to geographical location, as the bulls in the current study all originated from the Southwest and Southeast regions of the United States as compared to all bulls originating from the Midwest in Wickware et al. [16]. Geographical influences of the microbiome of *T. foetus*-infected bulls will be difficult to investigate due to disease prevalence in the Southwest and Southeast regions as compared to the Midwest. [24]

The higher alpha diversity observed in our study of *T. foetus*-infected bulls is consistent with findings in infected heifers. This finding underscores the dynamic nature of microbial communities in response to pathogenic challenges, with significant implications for understanding reproductive health and disease management in cattle. In a 2017 study evaluating the effect of *T. foetus* on the vaginal bacterial microbiota of heifers, Salles-Martins found that infected heifers had a significantly higher alpha diversity than the control group [25]. Salles-Martins also observed that most bacterial families detected in the infected heifers were also in the control groups, just at lower abundances. The author goes on to propose that this finding demonstrated the community dynamic and proliferation behavior of commensals in unstable environments is caused by host–parasite interactions [25]. While in our current study we did not have a bull control group, when we compared it to the Wickware et al. uninfected bull study, our study shows that the *T. foetus* infection status animals had a higher alpha diversity, as well as agreeing with Salles-Martins’ study findings. The healthy bulls of the Wickware et al. study had an estimated species richness range from 10 to 440, whereas in our current study, the estimated species richness ranges from 237 to 2794. This likely suggests that similar to what occurs in infected females, infected bulls too have an unstable environment due to host–parasite interactions leaded to a dysbiosis of common commensals of the reproductive organs.

## 5. Conclusions

This study characterizes both the preputial and seminal microbial community of bulls chronically infected by *T. foetus*. The results of this study support the hypothesis that *T. foetus* infection may lead to shifts in the reproductive microbiome similar to what was noted in the female and that the disease does cause a dysbiosis as compared to healthy, uninfected bulls. These findings may impact future treatment options that would allow infected bulls to clear the infection and thus be maintained as herd sires. However, future studies need to be carried out to determine the exact reproductive impact of the disease, for example, looking at longitudinal sampling before and after infection. There was no statistical difference in the collection device used, which necessitates the need for future studies with larger animal populations. In addition, the use of comparative groups between infected and non-infected animals housed in the same environment will allow for better comparison and the ability to determine negative impacts in infected animals. Furthermore, future studies can survey veterinarians and determine which collection device is the easiest to use, most cost effective, and is preferred for taking diagnostic samples. This research is an important first step in describing the microbiome in the face of infection with *T. foetus* so that we may begin to understand the shift of the reproductive microbiome in the face of disease so that we can both better understand the disease process and work towards identifying better treatment and control measures.

## Figures and Tables

**Table 1:** Relative abundance difference between guarded swabs and dacron swabs. Statistical testing was done using T-test and level of significance was considered at *p*-value ≤ 0.05. “GRD” = guarded swab, “SWB” = dacron swab.

GRD-SWB			
Species	GRD (Mean ± SD)	SWB (Mean ± SD)	*p*-Value
*Comamonas kerstersii*	0.0006 ± 0.0006	9.8552 ± 3.2686	0.019
*Parabacteroides merdae*	0.0003 ± 0.0003	6.1102 ± 0.0001	0.011
*Alistipes finegoldii*	0.0001 ± 9.6366	2.7594 ± 5.8049	0.046
*Sphingobium xenophagum*	5.2233 ± 6.9890	3.9421 ± 8.7706	0.045
*Alistipes indistinctus*	3.9421 ± 5.4694	0 ± 0	0.037
*Selenomonadales bacterium Marseille-P2399*	2.5623 ± 3.3333	9.8552 ± 3.2686	0.034
*Alistipes shahi*	1.9710 ± 2.4152	1.9710 ± 6.5372	0.037
*Streptosporangiaceae str. KACC 20141*	0 ± 0	6.8986 ± 1.0021	0.045
*Rhodococcus fascians*	0 ± 0	3.9421 ± 5.4694	0.037
*Eubacterium* sp. *SN18*	3.9421 ± 5.4694	0 ± 0	0.037

**Table 2:** Relative abundance difference between pizzle sticks and guarded swabs. Statistical testing was done using T-test and level of significance was considered at *p*-value ≤ 0.05. “PIZ” = pizzle stick, “GRD” = guarded swab.

PIZ-GRD			
Species	PIZ (Mean ± SD)	GRD (Mean ± SD)	*p*-Value
*Clostridiales bacterium FK041*	0.0003 ± 0.0004	0.0023 ± 0.0028	0.041
*Sphingomonas paucimobilis*	1.0840 ± 1.9218	0.0006 ± 0.0008	0.039
*Campylobacter sputorum*	0.0002 ± 0.0002	0.0001 ± 9.5161	0.044
*Arthrobacter citreus*	3.2522 ± 4.2581	0.0001 ± 0.0001	0.049
*Bacteroides plebeius*	1.7616 ± 4.9826	0.0001 ± 0.0001	0.020
*Brevundimonas vesicularis*	8.1306 ± 2.2996	0.0001 ± 0.0001	0.027
*Campylobacter jejuni*	0 ± 0	3.2547 ± 4.0562	0.023
*Paenibacillus hunanensis*	0 ± 0	7.6871 ± 9.5799	0.023
*Aerococcus urinaehominis*	0 ± 0	2.4638 ± 3.6631	0.049
*Sphingobium xenophagum*	4.0653 ± 5.6106	5.2233 ± 6.9890	0.045
*Bacteroides helcogenes*	7.5885 ± 5.0183	7.8842 ± 1.0938	0.006
*Selenomonadales bacterium Marseille-P2399*	0 ± 0	2.5623 ± 3.3333	0.028
*Anoxybacillus toebii*	0 ± 0	9.8552 ± 1.2317	0.024
*Legionella* sp. *LHG-1BW4*	1.3551 ± 3.8328	1.5768 ± 2.0775	0.046
*Bacterium YE57*	2.4391 ± 2.1487	2.9565 ± 7.0104	0.026
*Eubacterium* sp. *SN18*	0 ± 0	3.9421 ± 5.4694	0.037

**Table 3:** Relative abundance difference between dacron swabs and pizzle sticks. Statistical testing was done using T-test and level of significance was considered at *p*-value ≤ 0.05. “SWB” = dacron swab, “PIZ” = pizzle stick.

SWB-PIZ			
Species	SWB (Mean ± SD)	PIZ (Mean ± SD)	*p*-Value
*Cronobacter sakazakii*	0.0003 ± 0.0002	2.4391 ± 4.9424	0.002
*Porphyromonas levii*	2.3652 ± 3.5566	0.0001 ± 0.0001	0.032
*Dietzia maris*	9.6581 ± 9.8102	2.1681 ± 2.9547	0.033
*Rugosibacter aromaticivorans*	3.2522 ± 3.0662	0 ± 0	0.005
*Alistipes finegoldii*	2.7594 ± 5.8049	0.0001 ± 0.0001	0.036
*Planococcus psychrotoleratus*	3.0551 ± 2.5106	5.4204 ± 1.1589	0.010
*Georgenia deserti*	1.1826 ± 1.3236	1.3551 ± 3.8328	0.028
*Bacteroides helcogenes*	4.9276 ± 1.3155	7.5885 ±5.0183	0.004
*Rhodococcus coprophilus*	8.8697 ± 1.0641	0 ± 0	0.019
*Stenotrophomonas chelatiphaga*	7.8842 ± 1.0938	0 ± 0	0.037
*Bacterium YE57*	3.9421 ± 1.0021	2.4391 ± 2.4187	0.033
*Alkalibacterium iburiense*	1.0840 ± 1.1875	1.3551 ± 3.8328	0.028
*Streptosporangiaceae str. KACC 2014*	6.8986 ± 1.0021	0 ± 0	0.004
*Rhodococcus fascians*	3.9421 ± 5.4694	0 ± 0	0.037

## Data Availability

The raw sequence data generated during this study are available in the NCBI repository under BioProject PRJNA988941.
