# Correction: Boggan et al. Describing the Reproductive Microbiome of *Tritrichomonas foetus* Chronically Infected Bulls and Diagnostic Collection Device Performance. *Animals* 2024, *14*, 2689

**DOI:** 10.3390/ani15101356

**Published:** 2025-05-08

**Authors:** SaraBeth Boggan, Babafela Awosile, Jennifer Koziol

**Affiliations:** School of Veterinary Medicine, Texas Tech University, 7671 Evans Drive, Amarillo, TX 79106, USA

## Missing Acknowledgements

In the original publication [1], the acknowledgement of Dr. Jason M. Fritzler was not included. The following acknowledgement should be added to the published paper: 

The authors acknowledge Jason M. Fritzler, for technical support during the conduct of the research.

The authors state that the scientific conclusions are unaffected. 

## Error in the Institutional Review Board Statement

There was an error in the original publication in the Institutional Review Board Statement. Due to an oversight in the proofreading stage, the incorrect date of obtained ethical approval was written—9 January 2022. The correct date is 1 September 2022. 

A correction has been made to the Institutional Review Board Statement in the back matter:

Ethics approval and consent to participate was obtained on 1 September 2022. Sample collection and animal handling were carried out following ethical guidelines approved by the Institutional Animal Care and Use Committee (IACUC) of the Texas Tech University with Protocol ID: IACUC 2022-1177.

The authors state that the scientific conclusions are unaffected. This correction was approved by the Academic Editor. The original publication has also been updated.
